# RFID trial: localization of non-palpable breast lesions using radiofrequency identification tags or wire

**DOI:** 10.1186/s12885-023-11190-w

**Published:** 2023-07-20

**Authors:** Hugo Veyssiere, Margot Dressaire, Raphaël Pete, Céleste Pinard, Ioana Molnar, Catherine Abrial, Angeline Ginzac, Xavier Durando, Marielle Tekath

**Affiliations:** 1 Université Clermont Auvergne, INSERM UMR 1240 « Imagerie Moléculaire et Stratégies Théranostiques », Centre Jean PERRIN, Clermont-Ferrand, France; 2 Division de Recherche Clinique, Délégation Recherche Clinique & Innovation, Centre Jean PERRIN, 58, Rue Montalembert, 63011 Clermont-Ferrand, France; 3 Centre d’Investigation Clinique, UMR501, Clermont-Ferrand, France; 4 Unité de Sénologie, Centre Jean PERRIN, Clermont-Ferrand, France; 5 Service de Radiologie, Centre Hospitalier Universitaire, Clermont-Ferrand, France

**Keywords:** Breast cancer, Non-palpable lesions, Patients comfort, Wireless breast localization, Radio-frequency tags

## Abstract

**Background:**

Breast cancer is the most frequently diagnosed cancer and the leading cause of cancer death in women. Approximately 50% of breast cancers are discovered at an early stage in patients for whom conservative surgery is indicated. Intraoperative localization of non-palpable breast lesions is generally accomplished using a hook wire to mark the area of concern under ultrasound or stereotactic localization. But this technique has several drawbacks (painful, stressful…). We propose the use of a wire-free breast lesion system using miniature radiofrequency identification (RFID) tags. This technique could improve patient comfort and surgical comfort for surgeons. We therefore propose a study to assess the interest of introducing the RFID localization technique at the Jean PERRIN comprehensive cancer center.

**Methods:**

This is a single-center prospective trial designed to assess the interest in introducing the RFID localization technique at the Jean Perrin center. It aims to show the superiority of the RFID technique in terms of patient tolerance compared to the gold-standard (hook wire). A sequential inclusion in time will be performed: 20 inclusions in the gold-standard group, then 20 patients in the RFID group before repeating the inclusion scheme. Any patient requiring preoperative localization will receive a senology consultation. The RFID tag will be placed during this consultation. The hook wire localization will be done the day before the surgery. Patients will fill out a Hospital Anxiety and Depression scale (HAD) questionnaire at the time of inclusion. They will then fill out a satisfaction questionnaire in 2 steps: during the placement of the device (RFID tag or hook wire) or during the postoperative consultation at 1 month. Radiologists and surgeons will fill out a questionnaire to evaluate the localization technique, respectively after the localization and surgery procedures.

**Discussion:**

The RFID study is the first study in France which specifically assesses the interest of the RFID localization in terms of patients comfort. Patient comfort is one of the key elements to take into consideration when managing patients in oncology and new technologies such as RFID tags could improve it.

**Trial registration:**

ClinicalTrials.gov ID; NCT04750889 registered on February 11, 2021.

**Supplementary Information:**

The online version contains supplementary material available at 10.1186/s12885-023-11190-w.

## Background

Breast cancer is the most frequently diagnosed cancer and the leading cause of cancer death in women with 58,500 new cases in France in 2018. Approximately 50% of breast cancers are discovered at an early stage in mainly young patients and for whom conservative surgery is indicated. Intraoperative localization of non-palpable breast lesions is generally accomplished using a hook wire to mark the area of concern under ultrasound or stereotactic localization.

However, this technique has several drawbacks. Indeed, it appears as a relatively painful and anxious procedure for patients. It can also be a source of difficulty for the surgeon, because only the entry point is visible: the orientation and length of the wire are assessed on a control image. Moreover, it leads to a time coordination obligation between radiologists and surgeons since the localization device cannot remain in place for more than 24 h. Finally, because the end of the hook wire comes out at one end of the breast and goes through the skin it can be associated with a risk of infection, breakage, accidental exposure to blood or wire dislodgement.

During the last decades, other localization techniques have been studied as alternatives to hook wire [1]. None of these alternatives has been able to prove its superiority compared to hook wire, which remains the gold-standard. A wire-free breast lesion system using miniature radiofrequency identification (RFID) tags appears as an interesting alternative (LOCalizer™ with CE marking and FDA labelling). It is a radio-opaque tag that emits a radiofrequency signal when excited at a specified wavelength. It is carried by a 12-gauge coaxial needle and can be positioned under ultrasound or stereotactic guidance. A receiver that registers the RFID number is available for radiologists and handheld reader displays the distance to the tag in millimeters (up to 6 cm) for surgeons. This technique aims to improve patient comfort and surgical comfort by increasing localization accuracy. Moreover, radiologists would no longer be dependent on the surgical schedule. The oblong shape of the tag should reduce the risk of accidental exposure to blood, and material breakage. Nevertheless, the cost of this technique remains significantly higher than the hook wire. Acceptance and changes in practices by radiologists and surgeons are necessary.

To date, few articles address radiofrequency localization, and most studies are of recent publication [2–5]. The main publications on radiofrequency localization deal with a small number of patients, without comparison to the gold-standard, and primarily assess safety, acceptance, and technique performance [2, 4, 6]. Only one American retrospective study implies a large number of patients (*n* = 503) and compares RFID localization qualities to the gold-standard [3]. They concluded that RFID tag localization is a possible alternative to hook wire and should be considered for non-palpable breast lesions. To date, no study has looked at the interest of the technique in terms of patient comfort. We therefore propose a study to assess the interest of introducing the RFID localization technique at the Jean Perrin comprehensive cancer center.

## Methods and study design

### Study objectives and outcomes

The primary objective of the RFID trial is to show the superiority of the RFID technique in terms of patient tolerance compared to the gold-standard (Table 1). It also aims to determine surgeons and radiologists perceptions of the RFID technique compared to gold-standard.

**Table 1:** Primary and secondary objectives

**Primary objective**	• To show the RFID technique superiority in terms of patient tolerance compared to the gold-standard
**Secondary objectives**	• To compare the surgeon’s perception of the localization by RFID to the gold-standard
	• To compare the radiologist’s perception of the localization by RFID to the gold-standard
	• To demonstrate the non-inferiority of the RFID technique over the gold-standard in terms of performance

### Study design

**Fig. 1:**
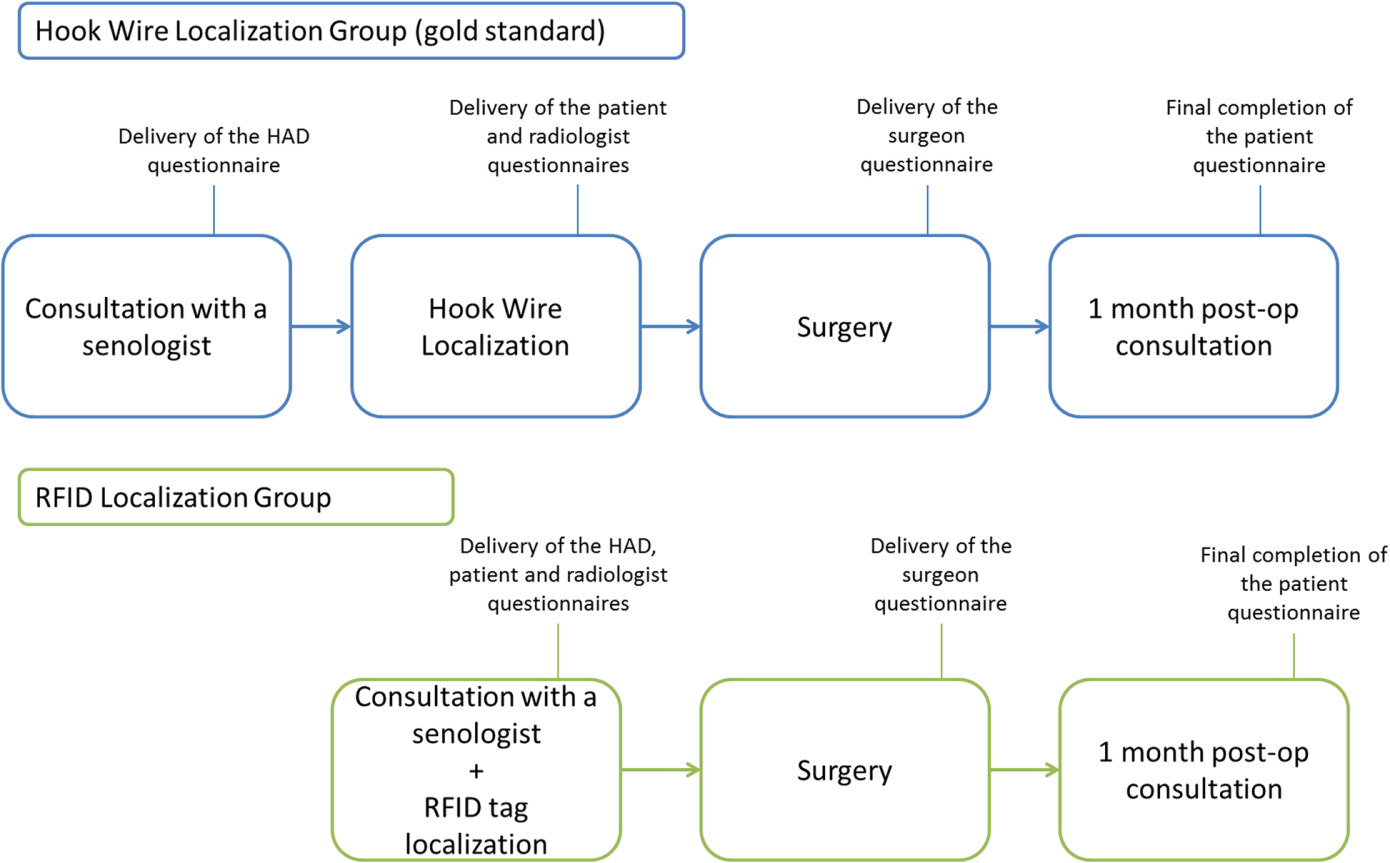
Design of the RFID study. The participation to the study will be proposed during a senology consultation to any patient requiring preoperative localization. The RFID tag and the hook wire will be respectively placed during the senology consultation and the day before the surgery. In the two groups, patients will fill out a Hospital Anxiety and Depression scale (HAD) questionnaire and then fill out a satisfaction questionnaire during the placement of the device (RFID tag or hook wire) and during the postoperative consultation at 1 month. Radiologists and surgeons will also fill out a questionnaire respectively after the localization and surgery procedures

This is a single-center prospective trial designed to assess the interest in introducing the RFID localization technique at the Jean PERRIN center. Study design is presented in Fig. 1.

Any patient requiring preoperative localization will receive a senology consultation and participation in the study could be proposed. A sequential inclusion in time will be performed: 20 inclusions in the gold-standard group, then 20 patients in the RFID group before repeating the inclusion scheme. Eighty patients will be enrolled.

The RFID tag or the hook wire will be respectively placed during the senology consultation and the day before or the day of the surgery. If the hook wire is placed the day before the surgery patients should stay in overnight.

As soon as they are included, patients will fill out a Hospital Anxiety and Depression scale (HAD) questionnaire to determine potential anxiety and depressive disorders.

The included patients will then fill out a satisfaction questionnaire in two steps:

- during the placement of the device (RFID tag or hook wire),
- during the postoperative consultation at 1 month

Radiologists and surgeons will fill out a questionnaire dedicated to them, respectively after the localization and surgery procedures.

### Primary and secondary outcomes

The primary outcome is the patient’s satisfaction score with the preoperative localization technique, evaluated by a visual analog scale (see patient questionnaire).

Secondary outcomes are:

- the patient’s pain score in relation to the preoperative localization technique, evaluated by a visual analog scale (see “patient” questionnaire)
- the score of the patient’s stress in relation to the preoperative localization technique (see patient questionnaire)
- the scores of the precision and the operative comfort associated with the device (see surgeons questionnaire)
- the visualization of the needle on ultrasound, the visualization of the device on ultrasound and on stereotaxic, and the ease of insertion of the device (see radiologists questionnaire)
- the occurrence or not of a migration of the localization device, the histological data with the presence of the lesion in the surgical specimen, the invasion of the margins and therefore the resulting rate of revision surgery.

### RFID Tag

The LOCalizer™ wire-free guidance system is designed by Hologic© to mark and guide to non-palpable breast lesions thanks to a radiofrequency identification (RFID) tag. Each tag has a unique identification number that is displayed on a reader and can be placed in the breast any time prior to or on the day of surgery.

### Questionnaires

We created the questionnaires to evaluate the patients’ satisfaction as well as the practical aspects of the localization technique from the radiologists and surgeons points of view (Additional file 1). Our questionnaires are partly based on validated models such as the Visual Analog Scale; to date, there is no fully standardized and validated model or questionnaire that would allow us to evaluate all the objectives of our study. Patients will also fill out a Hospital Anxiety and Depression scale (HAD) questionnaire [7].

### Patient selection

Inclusion and exclusion criteria are presented in Table 2. Patients over 18 year’s old requiring conservative surgery for an histologically proven non-palpable breast lesion (fibroadenomas, papillomas, atypical lesions and neoplasia) will be included.

**Table 2:** Inclusion and non-inclusion criteria

**Inclusion criteria**	• Female over 18 years old • Requiring conservative surgery for an histologically proven non-palpable breast lesion (fibroadenomas, papillomas, atypical lesions and neoplasia) • Speaking and understanding French • Patients referred to senology for localization of non-palpable breast lesions by surgeons • Affiliated to the French Social Security System • Able to give informed consent
**Non-inclusion criteria**	• Multiple breast lesions • Patients with breast neoplasia during pregnancy • Persons deprived of liberty or under guardianship or incapable of giving consent • Refusal to participate

The estimated duration of patients’ enrolment is 1 year and 6 months: 80 patients will be enrolled and followed from the inclusion until one month after surgery. A sequential inclusion in time will be performed: 20 inclusions in the control group (gold-standard) during 3 months, then 20 patients in the “RFID group” for the following 3 months with an interim statistical analysis, before repeating the inclusion scheme. When patients consent to participate in the study, they are informed of the device they will receive. Patients of the hookwire group are convened the day before the surgery to install the device. Patients with the RFID tag have the device installation at least one week before their surgery.

### Recruitment and consent to participate

Eligible patients will be offered the opportunity to participate in the study by their radiologist or their surgeon. Patients who agree to participate in this study will provide oral consent for enrolment and the investigator will sign a document demonstrating that the patient has been informed and has agreed to participate. Data obtained will be retained with consent, and any reasons given for withdrawal will be recorded. Participants can withdraw at any time.

### Sample size calculation

The satisfaction score is a continuous score taking values between 0 and 10 points. To be able to show a minimum effect size of ¾, corresponding to a difference of about 23% of the overall score, for an estimated standard deviation of about 1.5 points, with a power of 80%, and considering a type I error rate of 5%, a number of 30 patients per group would be necessary. To take into account a rate of non-matchable patients of about 20%, as well as a rate of non-evaluable patients of about 10%, 40 patients will be included in each group, for a total of 80. Given the lack of preliminary data, and the uncertainty around the assumptions used for sample size calculation, an interim analysis will be performed after approximately half of the initially planned number of subjects has been included. The purpose of the interim analysis is to re-estimate the number of subjects needed based on the distribution of scores, in order to ensure sufficient statistical power of the study. Since no early ending is planned after the interim analysis, the alpha risk will not be adjusted, and the formal comparison between the two groups by hypothesis testing will be done only at the final analysis.

### Data collections

Data collected are the patient’s age (month and year of birth), pathology, clinical and molecular characteristics of the tumour on biopsy and surgical specimen, the size of the target lesion during clinical examination, at mammography and ultrasound, and responses to questionnaires. Data collected will be pseudonymized. Thus, study data will not contain any names or other personal identifiers such as addresses. Patients included in the trial will be identified by a code specific to this trial. Investigators will have access to the correspondence table between the patient’s last name, first name, date of birth and the code assigned in the trial.

### Statistical analysis

#### Primary analysis

The main analysis consists of comparing the patient satisfaction scores between the two groups, RFID vs. gold standard, and, if the difference in mean scores is clinically significant (difference of about 20%), to test whether it is statistically significant using Student’s t-test or a non-parametric test depending on the nature of the data. A propensity score matching will be performed beforehand to compensate for the non-randomized design of the study. We will include in the calculation of the propensity score the anxiety measured by the HAD scale at inclusion, the only measurable confounding factor we estimate being related to the primary outcome.

#### Secondary analysis

The secondary analysis consists of the analysis of the global score values, and the comparison between the two study groups, of the patient questionnaires (with matching or adjustment for baseline anxiety values), the surgeons and the radiologists, using the Student’s t-test (/ non-parametric test) or the chi-square test (/ Fisher’s exact test), depending on the nature of the data. If a statistically significant difference between groups is found, a post-hoc analysis will follow on the questionnaire items, with multiple testing corrections.

Data on localization quality will also be analysed and compared between groups using one-tailed tests to ensure that they do not indicate an inferiority of the experimental device.

### Trial status

As of this day, 64 patients have been recruited in the RFID trial. Participant recruitment began on 12 May 2021 and is expected to finish in November 2022. The approved protocol is version 10, 26/04/2022.

### Patient and public involvement

Neither patients nor the public were involved in the design of this research.

### Ethics approval and dissemination

The RFID trial has been approved by an ethics committee (Ile de France XI) on May 2021 (ID-RCB number: 2021-A00281-40). It is conducted notably in accordance with the Declaration of Helsinki and General Data Protection Regulation (GDPR). Study data and findings will be published in peer-reviewed medical journals. We plan to present the study and all data at national congresses and conferences.

## Discussion

Preoperative breast localization using hook wire remains the gold standard technique. But it has restrictive time requirements for surgeons and radiologists. It also causes patient discomfort and anxiety. Thus, this has led to finding and developing wire free alternatives. In this study, we focus on the wire free localization using RFID tag. Wazir et al. have shown that wireless localization using RFID is an effective and time-efficient alternative to wire guided localization [4]. These RFID tags appear as a great alternative since they could improve patients comfort and reduce the restrictive scheduling requirements imposed by the gold-standard localization.

Moreover, the RFID study is the first study in France which specifically assesses the interest of the RFID localization in terms of patients comfort. Patient comfort is one of the key elements to take into consideration when managing patients in oncology and new technologies such as RFID tags could improve it.

However, the RFID technique has a major drawback: it remains more expensive than the hook wire technique. Nonetheless, in the long term, a generalization of the technique in comprehensive cancer centers and hospitals could allow a cost reduction.

## Supplementary Information

**Additional file 1.**

## Data Availability

Not applicable. This manuscript does not contain any data.

## Abbreviations

HAD: Hospital Anxiety and Depression scale
RFID: Radiofrequency identification
