# Supplementary material for: RFID trial: localization of non-palpable breast lesions using radiofrequency identification tags or wire
Source: BMC Cancer. 2023 Jul 20;23:679. doi: 10.1186/s12885-023-11190-w (PMC10357842; doi:10.1186/s12885-023-11190-w)
Supplement: Supplementary file 1 — Additional file 1. [file 12885_2023_11190_MOESM1_ESM.zip › Patient questionnaireR2.docx]

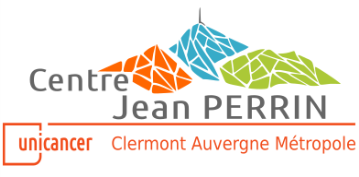


**RFID trial: Localization of non-palpable Breast Lesions**

**using radiofrequency identification Tags or Wire**

***Patients ‘ questionnaire***

***Date completed:***

***Inclusion number:***

***Patient's date of birth (month and year):***

For each of the questions below, please answer by drawing a clearly visible perpendicular line on the visual scales (in blue).

1. Can you rate the level of pain you felt when the localization device was placed?

Worst pain imaginable

Absence of pain

1. Can you rate the level of pain you experienced between the placement of the localization device and the surgery ?

Worst pain imaginable

Absence of pain

1. Can you rate the level of stress (or anxiety, tension) you felt when the localization device was placed ?

No Stress

Highest possible stress

1. Overall, how would you define your satisfaction with the localization technique ?

Fully satisfied

Not at all satisfied

Comments :
