# Supplementary material for: RFID trial: localization of non-palpable breast lesions using radiofrequency identification tags or wire
Source: BMC Cancer. 2023 Jul 20;23:679. doi: 10.1186/s12885-023-11190-w (PMC10357842; doi:10.1186/s12885-023-11190-w)
Supplement: Supplementary file 1 — Additional file 1. [file 12885_2023_11190_MOESM1_ESM.zip › Radiologist questionnaireR2.docx]

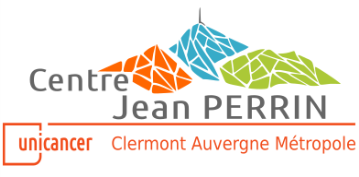


**RFID trial: Localization of non-palpable Breast Lesions**

**using radiofrequency identification Tags or Wire**

Radiologists’ questionnaire

***Date completed:***

***Inclusion number:***

***Patient's date of birth (month and year):***

Please specify the duration of the procedure (in minutes):

- Please check the localization guidance type :
- Ultrasound
- Stereotactic

*Please answer the following questions by checking the most appropriate answer.*

- How would you judge the visualization of the needle in ultrasound :

| - Very poor | - Poor | - Average | - Good | - Excellent |
| --- | --- | --- | --- | --- |

- In ultrasound, how would you rate the visualization of the device (hook wire tip/RFID tag):

| - Very poor | - Poor | - Average | - Good | - Excellent |
| --- | --- | --- | --- | --- |

- Did a migration occur during the device placement?
  - No
  - Yes, if yes …….. cm
- How would you rate the overall ease of placement of the localization device?

| - Very easy | - Easy | - Intermediate | - Hard | - Very Hard |
| --- | --- | --- | --- | --- |

*Comments :*
