# Supplementary material for: RFID trial: localization of non-palpable breast lesions using radiofrequency identification tags or wire
Source: BMC Cancer. 2023 Jul 20;23:679. doi: 10.1186/s12885-023-11190-w (PMC10357842; doi:10.1186/s12885-023-11190-w)
Supplement: Supplementary file 1 — Additional file 1. [file 12885_2023_11190_MOESM1_ESM.zip › Surgeon questionnaireR2.docx]

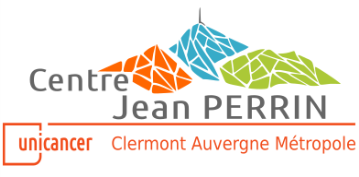


**RFID trial: Localization of non-palpable Breast Lesions**

**using radiofrequency identification Tags or Wire**

Surgeons ‘s questionnaire

***Date completed:***

***Inclusion number:***

***Patient's date of birth (month and year):***

Please specify the duration of the procedure (in minutes):

*Please answer the following questions by checking the most appropriate answer.*

- How would you rate the accuracy of the localization technique ?

| - Very poor | - Poor | - Average | - Good | - Excellent |
| --- | --- | --- | --- | --- |

- How would you rate the surgical comfort ?

| - Not at all comfortable | - Comfortable | - Very comfortable |
| --- | --- | --- |

- For the localization by hook wire, did an accidental exposure to blood occur?
  - Yes
  - No
- Did a dislodgement of the localization device occur?
  - Yes
  - No

*Comments :*
